# “When you are alone you have a narrow mind, but when you are with others you think broader into the other aspects”. A qualitative study on the role of sense of belonging and mattering in attempted suicide in Uganda

**DOI:** 10.1080/17482631.2024.2424012

**Published:** 2024-11-02

**Authors:** Birthe Loa Knizek, James Mugisha, Eugene Kinyanda, Julia Hagen, Heidi Hjelmeland

**Affiliations:** a Department of Mental Health, Norwegian University of Science of Technology, Trondheim, Norway; b Faculty of Social Sciences, Department of Social Work and Social Administration, Kampala, Uganda; c Medical Research Council/Uganda Virus Research Institute & London School of Hygiene and Tropical Medicine, Uganda Research Unit, Entebbe, Uganda; d Department of Mental Health Work, NTNU Samfunnsforskning AS (NTNU Social Research), Trondheim, Norway

**Keywords:** Suicide attempt, belonging, mattering, Uganda, culture

## Abstract

**Introduction:**

Suicide is globally a severe problem with an estimated 700.000 deaths annually. Six of the 10 countries with the highest suicide rates worldwide are in Africa, though, reliable statistics are scarce.

**Method:**

In this qualitative interview study in Uganda, we analysed the stories of 16 people admitted to hospital following a serious suicide attempt. We focussed especially on each person’s decision process towards their resolution to attempt suicide.

**Findings:**

Despite the huge heterogeneity of the narratives, we could identify problems regarding the sense of belonging and mattering in all the stories. Both the sense of belonging and mattering have been related to suicidal behaviour in earlier theories, but they were never studied together or under consideration of the influence of this specific cultural context. We found that the participants’ sense of belonging and mattering to a large degree was influenced by their traditional communalistic context with a worldview where the line between the natural and spiritual world was blurry.

**Conclusion:**

This kind of knowledge could be a valuable source for health professionals in their treatment of suicidal persons; it could direct their approach to the core of each person’s relational problems and meaning-making, which is crucial for their decisions with regard to suicide.

## Introduction

Globally, suicide is one of the leading causes of death. WHO (2021) estimates that 700.000 people take their lives annually, which means that one of every 100 deaths is due to suicide. Seventy-seven percent of the suicides occurs in low- and middle-income countries (WHO, 2023). In the African region, the suicide rate is estimated to be around 11/100 000, which is higher than the global average of 9/100 000 people (WHO, 2022). Six of the 10 countries with the highest suicide rates worldwide are in Africa (U.S. News, 2023; WHO, 2022). The suicide attempt rate is estimated to be around 20 times higher than the suicide rate. Bearing in mind that reliable data are scarce, WHO states that “globally, the availability and quality of data on suicide and suicide attempts is poor. It is likely that underreporting, and misclassification are greater problems for suicide than for most other causes of death» (WHO, 2022).

Several factors might contribute to both underreporting and misclassification. Governments’ underinvestment in adequate mental health provision must be taken into consideration. For example, in Uganda the tackling of the mental health problems of people seems overwhelming due to a severe shortage of mental health professionals and primary care providers’ reluctance to deal with mental health care (Blain, 2014; Kaggwa et al., 2022; Wagner et al., 2014). WHO Regional Director Moeti stated that “*Suicide is a major public health problem and every death by suicide is a tragedy. Unfortunately, suicide prevention is rarely a priority in national health programmes*” (WHO, 2022). He also argues for a radical change as mental health is integral to wholesome health and well-being, and far too many people in their region who need help for mental health problems do not receive it.

In addition to an under-resourced health system, the normative context must be considered as a factor contributing to underreporting and misclassification. First, Uganda’s Law criminalizing attempted suicide (Penal Code Act Chapter 120 of the Laws of Uganda) might scare people from reporting suicide attempts (Hjelmeland et al., 2012). Second, all religions condemn suicide, and since most Africans are very religious (Mbiti, 2006) and integrated in a religious community, this might also contribute to people refraining from reporting suicidal behaviour. The interdependent community structure and communalism also are still dominant and with this comes a perception of suicide as a strong taboo (Mugisha et al., 2014, 2018; Ndosi, 2006). Suicide and suicidal behaviour not only affect individuals but have heavy consequences for entire families that risk expulsion from the community (Mugisha et al., 2011; Osafo et al., 2011). In a close interdependent context, expulsion is extremely severe, and isolation follows both for the suicidal individual and his/her family.

Because of the severe consequences that suicidal behaviour has for individuals and entire families, prevention efforts must take into consideration threats to the well-being of individuals. Hence, the phenomena of belonging and mattering are of interest as both are components of an individual’s feeling of loneliness, which has been declared a global threat (The Guardian, 2023-11-16). Belonging and mattering are closely related as “ … *both derive from the need to validate our identity, and our existence, through personal affirmation*” (Prilleltensky & Prilleltensky, 2021, p. 30). Baumeister and Leary (1995) equalized the need for food and belonging as fundamental human motivations, necessary both for survival and flourishing. Prilleltensky and Prilleltensky make the compelling formulation that “*just as belonging is a powerful tonic for well-being, exclusion is toxic for health*” (2021, p. 31). Belonging thus can be viewed as a person’s feeling regarding a specific group. Prilleltensky and Prilleltensky (2021) also claim that a need for dignity, together with a sense of belonging and secure attachment, is an essential part of feeling valued that is fundamental for our well-being.

The phenomenon of mattering has a long story, but as a concept it was brought forward by Rosenberg and McCullough (1981), who focused on the significance to self-esteem of being valued by others. In a study on the relationship between mattering and suicidal ideation, Elliot et al. (2005) clarify why mattering is important: “*If one does not matter, this is a self-evident sign that one is not integrated into the social order, and there is little incentive to become so integrated*” (p. 236).While belonging is an individual’s feeling of being integrated in a group, mattering is an individual’s perception of self-worth based on the experiences and feelings caused by other persons’ behaviour. If other people attend to a person and invest themselves in him/her, or seek to him/her for resources, Elliott et al. (2004) regard this as mattering, and it contributes to the individual’s self-esteem. The perception or feeling of mattering relies on three sources: (1) awareness (i.e., the feeling of being of interest and noticed by others), (2) importance (i.e., the feeling that others are concerned about you), and (3) dependence (i.e., the feeling that others rely and depend on you) (Cha, 2016; Dixon, 2018; Elliott et al., 2004; Rosenberg & McCullough, 1981).

Whether mattering and sense of belonging are separate constructs, is an ongoing controversy. While perceptions of mattering occur through individuals’ interpretations of both the quality and quantity of other persons’ behaviours towards them (Dixon Rayle, 2005), a sense of belonging is thought to be more oriented towards a group. Mattering thus most often occurs between two persons, while belonging is conceptualized as belonging to groups (Baumeister & Leary, 1995; Dixon Rayle, 2005). The relational aspects of an individual’s self-perception thus are central. Flett (2022) describes that a person’s perception of whether (s)he matters or not is a question of life and death for some individuals. For example, studies have indicated that people’s mattering might have been implicated in suicide attempts and suicides (Addington & Mancuso, 2009; Nolle et al., 2012). Others have linked the perception of mattering with suicidal behaviour (Holden et al., 2018) and suicidality (Elliot et al., 2005; Joiner et al., 2009; Milner et al., 2016), which makes mattering a central phenomenon in the study of suicide attempts.

Like mattering more recently has been linked to suicidality, a sense of belonging has been linked to suicidal behaviour since Durkheim in 1897. However, in a systematic review of studies on suicidality and belonging, Hatcher and Stubbersfield (2013) conclude that there is only a weak association between suicidality and sense of belonging, partly because of culturally insensitive measures, where it seems hard to distinguish sense of belonging from other measures of social support or loneliness. They advocate for alternative ways of looking at connectedness, as well as for studies from a clinical setting as they do not find earlier studies culture sensitive enough.

As our study took place in Uganda, we necessarily had to consider an African worldview as the background on which to understand people’s acts. In an African worldview, the individual is seen differently than in the Western view. According to Verhoef and Michel (1997), the individual, object, person, or concept is not seen as occurring in itself, since entities within the African ethos only exist within relationships: “*Unlike the Western view of person as individual, the concept of person in the African world view is first and most importantly that of community. One does not focus on oneself as a distinct entity, but in relation to others*” (Verhoef & Michel, 1997, p. 396). The communalistic worldview even goes beyond life and reaches into the spiritual world.

Mugisha et al. (2018) have pointed out that Ugandans struggle both in life and in death to have a sense of belonging to the community and the spiritual world. The struggle is based on the cyclical African worldview, where those being dead continue living as ancestors in a different world with supernatural powers and can be reincarnated (Ekore & Lanre-Abass, 2016). Ancestors, who died naturally after a long life, are believed to have the power to bless or to curse, as well as to give or take life (Ekore &Lanre-Abass, 2016). As a result, people traditionally are buried together for generations to maintain their sense of belonging also beyond death. If death occurs unnaturally or even is condemned, like in suicide, the deceased person will be buried separately and without the traditional ceremonial burial (Mugisha et al., 2018). In this case, the deceased person is believed to end up as a wandering ghost and represent a danger for the still living (Ekore & Lanre-Abass, 2016). Mattering and belonging in both life and death seem to be of existential importance in this setting and are phenomena that must be considered when studying suicidal behaviour. The aim of the study was to explore the experiences of persons admitted to hospital following a suicide attempt in Uganda, with a particular focus on aspects related to belonging and mattering. Our research questions were: What is their story behind their suicide attempts? How does their story relate to the concepts of belonging and mattering?

## Method

We employed a qualitative design with semi-structured interviews. Our approach was inspired by Interpretative Phenomenology (Pietkiewicz & Smith, 2012), which underlines the advantage of semi-structured interviews to engage in dialogues with sufficient space and flexibility that allow unexpected material to arise. This approach is fruitful for exploring new aspects of suicidal behaviour.

### Participants

We interviewed persons admitted to hospital following a suicide attempt in the interdependent society of Uganda. We invited them to tell their story about what led them to try to take their own lives and whether they could identify possible resources. In our presentation, we chose the term suicide attempt, which is in accordance with the participants’ definition. We interviewed 16 persons, 13 men and 3 women (18+ years; age range 19–65, mean age 33) The basic inclusion criterion was that the person could tell his/her story in either English or Luganda. The participants belonged to different ethnic groups: Munyankole, Mutoro, Mugishu, Baganda, Alur, Lugbara, Alugbar, or Bafumbira. All were Christian but belonged to different branches. Their level of education ranged from primary 2 to university degree, but the majority were well-educated. Most of the participants were either unemployed or did random jobs, a few worked as craftsmen or farmers, one as a prostitute, and one as a tutor. The majority had attempted suicide more than once and one person had 7 previous suicide attempts. The methods were hanging, stabbing, drowning, burning, jumping from heights, headbanging into a wall, ingestion of pesticides or overdose of medication, or purposive alcohol consumption for attainment of death. At the time of the interview, all participants following a suicide attempt were hospitalized in a forensic, addiction, or other clinical ward.

### Setting

The interviews were carried out at Mulago National Referral Hospital and Butabika National Referral Psychiatric Hospital in Kampala (capital of Uganda) and Entebbe District Hospital (Entebbe). It is important to underline that attempted suicide still is considered criminal by law in Uganda (Penal Code Act. Chapter 20 of the Laws of Uganda). Religions condemn suicidal behaviour harshly and so do the strong traditions, which deem this kind of behaviour a taboo (Mugisha et al., 2014; Ndosi, 2006). This normative background influences both patients’ (Mugisha et al., 2011) and professionals’ (Knizek et al., 2012) attitudes and behaviour.

### Data collection

The interview guide comprised a narrative and a problem-focused part. To get the most valid information about the person’s understanding of the process, we initially invited them to tell their story. The narrative was followed with questions about their intentions, emotions, any abuse history, and religious life (if not covered in the participants’ narrative). Interview questions were for example: Can you please describe your feelings before the suicide attempt/self-harm? Can you say something about why you tried to kill/harm yourself? Would you describe yourself as religious? Is there any history of alcohol/drug abuse in your family? How did your family and close friends treat you when they found out that you had harmed yourself/attempted to kill yourself?

Three experienced Ugandan clinicians (the two Ugandan co-authors among them) conducted the interviews shortly after the suicidal act. All interviews were recorded and transcribed verbatim (and translated into English in the cases where the interview was conducted in Luganda).

### Data analyses

The first author analysed the data by means of Interpretative Phenomenological Analysis (IPA; Smith et al., 2021). This involved a detailed examination of the participants’ lifeworld, i.e., his/her personal experience and perception of any given event (Smith & Osborn, 2003), which in this case is a suicide attempt. IPA is carried out through seven steps: 1) reading and re-reading the interview transcripts, 2) taking initial notes, 3) developing emergent themes, 4) searching for connections among emergent themes, 5) moving to the next case, 6) looking for patterns across cases, 7) using theories as a lens for a deeper understanding (Smith et al., 2021). After the initial analyses, the themes were discussed among all authors, revised, and discussed again until consensus was reached. To ensure trustworthiness we employed different strategies as mentioned by Ahmed (2024): The credibility component was ensured by means of prolonged engagement with the participant, helping us to gain insight into history and reflections. Reflexivity was pursued by the team of authors from different cultures to contribute to richer and more nuanced understanding of the phenomenon under study. Transferability is based on thick descriptions allowing others to decide the usefulness of the study. Through the stories, we give a context to our findings, thus making the findings more transparent for others. The methodological descriptions enhance the dependability, while the confirmability of the interpretations was ensured through the constant discussions within the team of authors.

### Ethical aspects

The study was approved by the necessary Regional Ethics Committee in central Norway (REK midt: Møre og Romsdal, Trøndelag), approval number 2012/2176 and the Ugandan National Council of Science and Technology (UNCST).

The topic of this study is sensitive, and the participants constitute a vulnerable group. The interviewer, consequently, continuously monitored the effect of the interview on the participants as recommended by Smith and Osborn (2003) and thus sought process consent as described by Kavanaugh and Ayres (1998). It was emphasized to the participants that they could decide to terminate the interview at any time and without consequences for themselves or their treatment at the hospital.

Informed consent was secured for each participant. In the following presentation, participants are referred to by gender and age only, for the preservation of their anonymity. A short debriefing session was included at the end of each interview where the participant was able to express his/her feelings about being interviewed on this topic and the interviewer assessed whether there was a need for follow-up after the interview. Such follow-up was deemed necessary in one case.

## Findings and discussion

The interviews revealed 16 very different stories behind the suicide attempts. All, however, circled around feelings of belonging and/or mattering or lack thereof. As indicated by the presented cases, mattering and a sense of belonging seem to be highly individual. This is in keeping with Flett who states: “*People differ not only in their feelings of mattering, they also differ in the need to matter and avoid feeling like they don’t matter”* (Flett, 2018, p. 68). The same could be said about the sense of belonging. However, in common for most of our participants seems to be a problem in either one or the other or both. Surprisingly, we also found that some participants had both a sense of belonging and mattering to some significant others, but still did not get their needs for belonging or mattering fulfilled. We did not find any participants perceiving that they mattered to others without a sense of belonging.

Our participants’ stories included different varieties of belonging and mattering and both the physical and spiritual realm. Our main themes mirror these complex dynamics and are (outlined further below): 1) Neither sense of belonging nor mattering, 2) Sense of belonging but not mattering, 3) Sense of belonging and mattering to the spiritual world. Within these themes, we found relational issues with living people, ancestors, as well as other supernatural beings.

### Neither sense of belonging nor mattering

In 2013, Hatcher and Stubbersfield stated that numerous studies have confirmed that belonging, both as a type of connectedness and social support, is a potential protective factor against suicidal behaviour at an individual level and possibly even at a public health level. Five of our participants felt neither a sense of belonging nor mattering and seem to have lost the protective shield of both belonging and mattering. However, their stories leading up to this were very different. All stated that their reason for having lost a sense of belonging was the way they were treated by other people. Most of them felt ostracized or mistreated by their family and they did not experience mattering to them.

An example is a young man in his twenties, who was dumped by his mother and grew up with numerous cousins at an aunt’s household. They abused him and reminded him all the time that he was dumped right from childhood. In addition, his father disowned him, which is devastating in this patriarchal and traditional community. He felt rejected by his relatives:

I was frustrated because the situation was so bad to the extent that the world had become useless whereby I had nowhere to stay, no job where I would earn some money for survival and whenever I went to my relatives, they would say that I had gone there to commit suicide so that I bring for them problems and so they would chase me away. (M, 26)

The family would always chase him away since he had attempted suicide several times before with the intent to «get rid of this world». He perceived not mattering to the relatives and was brought up in a way where he repeatedly was reminded about his worthlessness. Furthermore, nobody else had given him the opportunity to develop a sense of belonging to them. According to Flett (2018), an individual who feels like (s)he does not matter is a person who has been unable to resolve or satisfy the inherent need to matter. Past life experiences will define the level of need to matter and if unresolved, the need can dominate this person’s thoughts, feelings, and behaviour. This young man is an example of those who has experienced being devalued by others for a long time and consequently lost their sense of belonging. Another young man, whose relatives had deprived him of all his properties and then rejected him, stated vividly:

Relatives do not look at me like I am a human being. All is now over. (…). Doctor, I was frustrated, I hated myself. I was frustrated because all people had rejected me. Whoever knows me, it reaches the time when he no longer knows me. All neglected me. Am alone as an individual. (M, 24)

Our participants reported abuse and/or neglect from relatives, spouses, parents, colleagues, or their entire community. Prilleltensky and Prilleltensky describe how exclusion generates feelings of *“ … inferiority, anger, humiliation, sadness, and shame. Feeling devalued and dehumanized are often the case … (…) What’s more, the victims tend to engage in self-defeating behaviors (…). Suicidal ideation and attempts have been documented”* (Prilleltensky & Prilleltensky, 2021, p. 39). In an interdependent environment, exclusion might be even more devastating than in an independent environment.

Most of the participants reported being ostracized without physical assaults, but some reported far more cruel circumstances. Like the young man in his twenties, who grew up without a father at the uncle’s place, while the mother fled from a forced marriage with the uncle. Later, when the uncle failed to be able provide his school fees, he was brought back to the mother, who had then re-married with a Muslim and as a Christian he could not stay with her. However, she paid his school fees until his last year at university, where she ran into financial problems. Until then, his problem was that he was pushed from one place to another without control or attachment and now had to provide for himself. Unfortunately, at this point he was approached by a stranger, who offered him a job in another country, but in fact abducted him and kept him as a prostitute in a remote place. He was locked up in a room and threatened severely. He was forced to have sex with both men and women, while being filmed. For two months he was drugged, forced to drink alcohol, and to take Viagra, as well as being exploited in a cruel way. At one point he managed to escape, and he tried to get help from several different institutions, but without success. In the meantime, he had become incontinent and had anal bleeding, which he tried to hide with toilet paper. He was sleeping outside and needed to drink alcohol to be able to sleep. In his darkest moment, he tried to call his mother, but it turned out that she had passed away without him having been told. Hence, he had been unable to bury her. Not to bury one’s parent is problematic in this context, since it meant he had been unable to pay respect to his mother, whom he had a connection to. This way, he had been ostracized once again. He had attempted suicide twice because he had lost hope in humanity and had not gotten justice for the evil that was done to him.

### Sense of belonging but not mattering

Seven of our participants seemed to have kept a sense of belonging to somebody, though they did not have the feeling of mattering.

For example, two of our participants (both men) felt that they could not live up to the community’s expectations. As a result, they felt excluded and alone, not mattering to anybody close to them. For example, a poor, young security guard (25) felt ashamed that he had nothing to give to the family or old friends and when he eventually managed to give them something, they were not grateful. By not appreciating his efforts, family and friends signalled to him that he did not matter to them. He tried to befriend colleagues, but they were from a different tribe, which caused difficulties in developing a sense of belonging with them. When he developed additional health issues, he decided that he was not worth living:

I felt so hope … I felt life was hopeless. I didn’t have any hope, though I still have a lot to do. First of all, I know I am alone, single, so I didn’t want to disturb my parents, or anything to disturb my people. I was a loser. I said let me take away the life, I go and relax. (M, 25)

In his statement, we can trace some ambivalence as he declares that he has a lot to do (referring to being the oldest brother carrying a lot of responsibility), though he has lost hope. He is very considerate and argues that he is without a spouse and did not want to disturb his parents or his people. His problem seems to be a sense of belonging to both parents and community, but feelings of not mattering as he was not allowed to contribute. He described his suicide attempt as an act of responsibility being the oldest brother, who had many people officially relying on him, but as they would not accept his efforts, his status was a problem. After the suicide attempt, he was afraid of being stigmatized and ridiculed and claimed that he, who had nothing, had no reason to live: “*Even the Bible says every tree which bears bad fruit should be cut down*”. In this case, the young man still believed in God and found an argument in the Bible for his suicide attempt, and maybe even for another in the future as his situation seemed worsened. Before, he did not matter to the people that he felt he belonged to, but now he is afraid of being excluded even more and stigmatized.

A young homeless woman experienced a similar problem. She had graduated from university and gotten a job, where she was bullied by a colleague. From there, her misfortune started, and she ended up sleeping in the streets, going from bar to bar and drink rather than eat. The lifestyle changed her appearances so much that she was embarrassed to return to the family:

Now, here life became so hard for me that the hair had changed its colour, legs and feet became swollen and eaten by jiggers and I knew the family where I am from. I said I cannot go back; they may look at me and fail to eat food or vomit. That’s how I came up with a plan of killing myself. (F, 29)

Her shame about her appearances was very strong and she decided rather to be dead than to show herself to the family. She felt that she brought shame over the family as she neither looked nor behaved like she came from a good family. She considered herself useless and could not see any reason to remain on this earth. She was tired of suffering and wanted it to end. Russel et al. (2019) stated that loneliness is the twin sister to uselessness. To consider oneself useless might be interpreted as a question of a perception of not mattering to others, while she still felt that she belonged to the family and tried to spare them from herself.

Another participant, who was struggling with his identity, was a young man in his early twenties. He was a university student. His father died and he was staying with his mother and a younger brother and two older female cousins. The family members were close and friendly with each other. His life had been fine, and he got everything he needed, but his father, who died of alcohol abuse, was absent. He thought the father was a fool and deserved to die, but now he had started drinking himself. His biggest problem was disappointment with his friends. When he still had money, he would spend them on his friends, whereas the friends declined to help him when he needed financial support from them. Consequently, he felt betrayed. He reported emotional pain, which he denied being related to the father, but more to the disappointments with his friendships:

He [Father] deserved the way he died. That was his problem. Because he had been even away from us. I spent years without seeing him. I only saw him on his death bed. He separated with my mother. So, I only saw him on his death bed. His thing does not bother me because am close to all my stepbrothers and sisters. His thing does not bother me. (…) Let him go. The friendship thing had a bigger bit. (M, 22)

Though he had a close family and made the impression of being somewhat immature, he missed a sense of belonging with friends, which made him insecure. His story included a very close relationship with a friend and a struggle for his welfare. The attachment seemed extraordinarily close, and he was devastated when he lost contact with him. His dominant problem was that he lost friends: “*Anyway the issue of letting friends go that is a different thing. The issue is in all my life, these friends you see just disappoint me*”. While the feelings of belonging and mattering were strong towards his remaining family, he longed to matter to men of his own age. Surprisingly, he did not seem to miss his father; he only blamed him and called him a fool. This is somewhat atypical and almost dramatic in this patriarchal context, where the division between life and death is blurry and the dead still are regarded active members of the family, who should be honoured (Ogungbemi, 2022). His story may indicate that he is struggling with his identity, circling around the issue of mattering to others.

### Sense of belonging and mattering to the spiritual world

According to Hatcher and Stubbersfield (2013), both belonging and mattering should have some protective value regarding suicidal behaviour but must be considered in light of individuals’ cultural contexts. In this specific context, we find a blurry line between life and death (Ogungbemi, 2022), which might indicate that mattering and belonging here reach beyond areas that Western theories describe. As traditional beliefs about afterlife are strong in this context, feelings of belonging were not necessarily reserved for living people but could include both ancestors and other spiritual forces. The spiritual realm could both be a threat and cause anxiety, but also serve as consolation and a possibility to meet loved ones again. Within this theme, we found narratives that illustrated both.

An example is a young man who stayed with his maternal grandmother, who had brought him up since childhood. However, his mother came to visit him before she died. His uncle had hurt him badly, when he told him that he had no clan or home and did not belong anywhere. To avoid his uncle’s recurrent insults, he moved away but did not enjoy life. He had no siblings, and he isolated himself. In addition, he was without a job that could occupy his mind, and he was overthinking and missed his mother:

Whenever I dream about my mother; I dream that she is calling me. So, when someone annoys me during daytime, suicide is the first thing that comes in my mind. I always think that when I die, I will meet her again. (M, 23)

His belief is that of an afterlife, where he can be reunited with his mother, whom he feels close to. Life and death are similar phenomena, though in different realms (Fobella & Mpeta-Phiri, 2023). Though he did not feel to matter or belong to the living family, he kept his bonding intact with the important ancestor, his mother. This thought seemed to be of consolation to him in his situation, where he had no feeling of belonging or mattering among the living people.

Another example is a teacher in his thirties with a diploma, who has worked for the government, but now was unemployed. He had a wife and three children and was a member of a structured drinking group with strong social bonds. They met at 7 pm every day and looked for each other if not present. The trigger for his two suicide attempts (both happening during the same night by hanging) was that he was provoked by a former colleague, who said things that he interpreted as witchcraft and a curse and consequently he felt threatened. This curse first targeted him personally and secondly his drinking group that he had a strong sense of belonging and mattering to. To protect the group, he decided to take his own life. In a third suicide attempt he tried to poison himself but was rescued by others. In his case, his belonging and mattering to the drinking group outweighed the relationship with his wife and children and the desire to protect others than the family was the strongest impetus for his suicide attempt. The perceived threat in this case was supernatural indicating the importance of the cultural, metaphysical beliefs making it a force that cannot be ignored. He consequently made use of spiritual measures to neutralize the threat. Before his suicide attempt, he had not been to church for three years, but now he used every opportunity:

On Friday I got to pray with Moslems and on Saturday I have to look for a nearby church with Seventh day and Sundays I try to resume my relationship with God through prayers. Since that day I have never missed prayer. (M, 39)

By shopping around, he hoped to safeguard himself and his drinking group who was significant to him. In a context where spirituality is regarded a very vital part of everyday life, his behaviour makes sense. Earlier research (Knizek et al., 2021; Mugisha et al., 2013; Osafo et al., 2013) has shown the importance of spirituality, especially regarding suicidal behaviour. In a context that is deeply religious and intertwined with the spiritual world, this factor must be considered:

In Africa, religion does not represent a philosophy of life that searches for ultimate meaning, as it does for many Western Christians today. Rather, it represents a view of life that acknowledges the existence of an invisible world, believed to be inhabited by spiritual forces that are deemed to have effective powers over one’s life. The spirit world is considered to be distinct but not separate from the visible world of human beings. One implication of such a worldview is that the invisible world is an extension of the visible one, and people’s social relations extend into it. Therefore, it is important for human beings to maintain a good relationship with this spirit world. (Ter Haar, 2009, p. 28)

We detected signs of this worldview in the consequences it had for several of our participants. The belonging to this invisible world could influence the relationships in the physical world as we saw in the example above. In the following example, a young man in his twenties stated that he had no problems with his family or community, but the problem seemed to be that his behaviour did matter to the ancestors, whose wrath he had aroused. He was married and had one child. He stated that his suicide attempt was due to hearing his ancestors’ voices:

I slept on the bed after eating my supper and in the middle of the night I heard voices from my ancestors because I heard them saying: “grandson do some sacrifices to us”. I woke up from sleep only to see am holding a knife cutting myself. (M, 27)

He then sought for help with the elders, who told him to go and dig on the father’s grave and now he feels healed. However, nobody was surprised that he had heard the ancestors’ voices as he used to be disturbed by these voices since he was young. In a ritual at the shrine following his suicide attempt, the traditional healer told him that he was supposed to be killed unless he repented his sins. The remedy was to go and drink some local alcohol and return to Catholicism. He now felt healed, but his wife was afraid of being alone in their house as she believed that the evil spirits might attack her as well. However, other people from the community were not afraid:*” … because some also have their spirits and small gods whom they worship. So, everybody there has their ancestral spirits, which they worship”*. (M, 27)

In contrast to the example before, where the threat was in form of an impersonal curse, the man in this case felt that he had issues with some forefathers, meaning persons from his own family in the spiritual realm. His story fits well with the beliefs of his community, hence nobody had any problems with his behaviour. In this community, it seemed natural combining Christianity and traditional beliefs: *«Religion is there, but you cannot neglect ancestral spirits. They get annoyed; you have to do all the rituals and also not forgetting God”*. (M, 27)

We found that questions of mattering and belonging in this context go beyond the physical world. but have equally strong consequences. We must consider a spiritual power influencing the natural world, sometimes as consolation or a threat and sometimes as a possibility. For example, one of our participants was a married woman, who was abused and scandalized by her husband. Her suicide attempts, she explained, were meant as attacks on her husband. As she was without any power in her real life, she supposed that in her death she could haunt him and make him miserable. In death, she could get power that had not been granted her in life. As death is not perceived as an end of life, but only as a transition into another realm, this seemed a good possibility to regain power and dignity.

As previously noted by Hatcher and Stubbersfield (2013), it seems that the existing knowledge on belonging and mattering is based on Western contexts. The stories of our participants seem to indicate that one must take another worldview into consideration to understand their misery fully as a basis of providing necessary help:

Medical professionals and caregivers working in African contexts also have an important role to play in understanding and respecting African cultural perspectives on death and afterlife, in order to provide culturally sensitive and appropriate care for the dying and the bereaved. (Fobella & Mpeta-Phiri, 2023, p. 66)

## General discussion

We found a huge variety of stories that all ended with suicide attempts. Abuse, neglect, ostracization, protection, revenge, anxiety of not fitting in or not being accepted are among the reasons that our participants mentioned for their suicide attempts. All these reasons are relational in nature, underlining the relationship between the suicidal individual and his/her significant others, either living or dead. Despite the heterogeneity of stories, we noticed common problems with a sense of belonging and/or mattering. Both a sense of belonging and mattering have been described as protective factors regarding suicide (Hatcher & Stubbersfield, 2013). Not surprisingly, we found that our participants struggled with one or the other, or both. Some of our participants had neither a sense of belonging nor a feeling of mattering, while others had kept a sense of belonging, but did not feel that they mattered. Others kept their sense of belonging but were afraid that they could not live up to expectations to them and therefore felt they did not matter. In this case, it was not other people’s behaviour that triggered the feeling, but their perception of failing to live up to own ideals. Still, this feeling of insufficiency seemed enough for them to feel lonely. Then, there were the participants who felt both a sense of belonging and mattering to their family, but where spiritual figures or forces seemed to exceed the family relationships in significance. To be recognized and matter to others consequently could encompass both living people, ancestors as well as other spiritual forces. The impact from the spiritual realm could be transformed into the participants’ perception of consolation, threat, or possible empowerment. Who and what might be regarded as most significant by the individual depends on the circumstances, contexts, as well on the individual’s attachments.

Among the living, it is important to consider both the intricate relationships and their hierarchic importance resulting from communalism in the interdependent culture, as well as the specific belief that the afterlife is a continuation of life in another realm (Fobella & Mpeta-Phiri, 2023). As stated by Caleb, one could extrapolate this worldview by saying “ *… that there is no real death in Africa if it is a continuation*” (Osore, 2021, p. 20). Consequently, communalism permeates the entire existence, whether it is in the physical or the spiritual realm. Dennis & Udo state that “*African individuals approach life in constant consciousness of an interconnection with some ‘other’, whose relation necessarily determine their behaviour—for they must consider the equal thriving of that ‘other’ in all they do*” (Dennis & Udo, 2021, p. 234). But they also admit that life has become increasingly challenging with present complexities due to being confronted with and pressured by higher and more complex socio-economic and political demands, which makes it more difficult to adhere to ancient moral laws (Dennis & Udo, 2021). In our study, we noticed that the fight over sparse resources sometimes resulted in rejection and ostracization of individuals, who lost both a sense of mattering and belonging and ended up isolated and lonely. Loneliness has been declared a global health threat, which was highlighted by the Guardian: “*The World Health Organization (WHO) has declared loneliness to be a pressing global health threat, with the US surgeon general saying that its mortality effects are equivalent to smoking 15 cigarettes a day*” (The Guardian, 2023-11-16). Loneliness might be a global threat, but due to different cultural contexts its meanings might differ. It seems therefore necessary to understand the different aspects of loneliness, which means problems regarding a sense of belonging and mattering. Those among our participants who had lost both clearly expressed their loneliness. Others, however, kept their sense of belonging, while their significant others signalled that they did not matter to them. This seems to be another kind of hurtful loneliness, where you feel that you are not accepted by those you feel that you belong to. In addition, a disharmonious relationship with ancestors or spiritual forces could result in an existential loneliness with severe consequences.

Communalism thus must be regarded as an underlying moral influence in the endeavour to understand suicidal behaviour considering mattering and belonging. The importance and impact of relationships was already indicated by the statement made by an elderly participant in the title: “*When you are alone you have a narrow mind, but when you are with others you think broader into the other aspects*” (M, 58). He expressed what has been shown by Sloan et al. (2017) that relationships are of overall importance for the meaning-making process of individuals. Belonging and mattering are central indicators of the individual’s perception of the relationships’ quality as indicated by Lambert et al.: “*To belong is to matter: Sense of belonging enhances meaning in life*” (Lambert et al., 2013, p. 1418). However, it seems that existing theories (Flett, 2018; Prilleltensky & Prilleltensky, 2021) do not cover all dimensions and aspects that we found in our study though universality has been claimed: “ *… it is likely that all human beings around the world maintain the innate desire and need to feel significant to others, to be needed and wanted, and to matter to them*” (Dixon, 2018, p. 2233). As indicated by Hatcher and Stubbersfield (2013), the universality claim might cover the need but miss the complexity of an individual’s sense of belonging and mattering in cultural contexts outside the Western world.

In this setting, we must emphasize the importance of both communalism and the perception of the circularity of life: Life does not start at birth and does not end with death. As expressed by Attoe: “*It [the worldview] is optimistic because it assumes a continuation of life after death, thus moving against the pessimism that comes with the idea of the finality of death*” (Attoe, 2023, p. 323). We found different examples of how this optimistic notion of the spiritual world could reach out and punish individuals by forcing them to sacrifice their lives, serve as a comfort for meeting loved ones again in the afterlife, or be a tool for revenge to haunt an assailant husband. In this specific cultural context, the concepts of belonging and mattering thus must be broadened to encompass both the traditional normative influence like communalism and even include spiritual figures. The influence of spirituality and religion on suicidal behaviour has been studied extensively (Akotia et al., 2014; Knizek et al., 2021; Mugisha et al., 2013; Osafo et al., 2013), but not from the perspective of mattering and belonging.

## Strengths and limitations

The strength of the study is the exploration of under-researched phenomena (belonging and mattering) regarding suicidal behaviour in an interdependent society, where belonging and mattering seem of great importance. A limitation of the study is that some of the interviews were performed by means of a translator and we consequently do not have full control over the exact expressions of the participants.

## Implications

As relationships seem crucial for the meaning-making of individuals and hence their decisions on their acts, the concepts of mattering and belonging seem fruitful to study further under consideration of a context’s specificity. This kind of knowledge could be a valuable source for health professionals in their treatment of suicidal persons; it could direct their approach to the core of each person’s relational problems and meaning-making, which is crucial for their decisions regarding suicidal behaviour. Questions on belonging and mattering could be a part of both anamnestic and therapeutic interviews and guide the professionals’ work.
